# 596 Elevated Cardiac Troponin I Level Within 72 Hours Correlated to Cardiac Dysfunction in Burned Patients

**DOI:** 10.1093/jbcr/irae036.230

**Published:** 2024-04-17

**Authors:** Carolina Segura, Isabel B Obias, Yash Ramgopal, Christopher G Richter, Sunskruthi Krishna, Suhaib Shah, Dalton Amador, Juquan Song, Georgiy Golovko, Amina E I ayadi, Steven E Wolf

**Affiliations:** University of Texas Medical Branch, Galveston, TX; University of Texas Medical Branch, Houston, TX; University of Texas Medical Branch at Galveston, Galveston, TX; University of Texas Medical Branch, Blocker Burn Unit, Galveston, TX; University of Texas Medical Branch, Galveston, TX; University of Texas Medical Branch, Houston, TX; University of Texas Medical Branch at Galveston, Galveston, TX; University of Texas Medical Branch, Blocker Burn Unit, Galveston, TX; University of Texas Medical Branch, Galveston, TX; University of Texas Medical Branch, Houston, TX; University of Texas Medical Branch at Galveston, Galveston, TX; University of Texas Medical Branch, Blocker Burn Unit, Galveston, TX; University of Texas Medical Branch, Galveston, TX; University of Texas Medical Branch, Houston, TX; University of Texas Medical Branch at Galveston, Galveston, TX; University of Texas Medical Branch, Blocker Burn Unit, Galveston, TX; University of Texas Medical Branch, Galveston, TX; University of Texas Medical Branch, Houston, TX; University of Texas Medical Branch at Galveston, Galveston, TX; University of Texas Medical Branch, Blocker Burn Unit, Galveston, TX; University of Texas Medical Branch, Galveston, TX; University of Texas Medical Branch, Houston, TX; University of Texas Medical Branch at Galveston, Galveston, TX; University of Texas Medical Branch, Blocker Burn Unit, Galveston, TX; University of Texas Medical Branch, Galveston, TX; University of Texas Medical Branch, Houston, TX; University of Texas Medical Branch at Galveston, Galveston, TX; University of Texas Medical Branch, Blocker Burn Unit, Galveston, TX; University of Texas Medical Branch, Galveston, TX; University of Texas Medical Branch, Houston, TX; University of Texas Medical Branch at Galveston, Galveston, TX; University of Texas Medical Branch, Blocker Burn Unit, Galveston, TX; University of Texas Medical Branch, Galveston, TX; University of Texas Medical Branch, Houston, TX; University of Texas Medical Branch at Galveston, Galveston, TX; University of Texas Medical Branch, Blocker Burn Unit, Galveston, TX; University of Texas Medical Branch, Galveston, TX; University of Texas Medical Branch, Houston, TX; University of Texas Medical Branch at Galveston, Galveston, TX; University of Texas Medical Branch, Blocker Burn Unit, Galveston, TX; University of Texas Medical Branch, Galveston, TX; University of Texas Medical Branch, Houston, TX; University of Texas Medical Branch at Galveston, Galveston, TX; University of Texas Medical Branch, Blocker Burn Unit, Galveston, TX

## Abstract

**Introduction:**

Severe burned patients are under a hyperadrenergic and hypermetabolic states. Without proper treatment, patients developed cardiac dysfunction and heart failure. Cardiac Troponin I (cTI) is released from cardiac tissue which is considered as having a high predictive value in cardiac injury. However, its biomarker potential in cardiac insult continues to be under investigation in burn patients. The purpose of this retrospective research study is to evaluate the role of cTI and its association to patients with burns.

**Methods:**

The patients' de-identified data was analyzed and collected from a national database. 8,092 patients 18 years old or older with burns who had cTI lab value were enrolled in this study. Patients were grouped by the cTI mean level within 72 hours including patients with high cTI levels at >0.3 ng/mL (n= 1,879 patients), and patients with normal cTI level less than at 0.04 ng/mL (n= 3,265). The cohorts were further stratified by less than 20% TBSA and >20% TBSA to replicate the severity of burns. The 30-days incidences of acute myocardial infarction (MI), sepsis, and mortality were observed after two cohorts were stratified into severe or mild burns. The cohorts were balanced using propensity score matching of covariates: age at index, sex, race, and ethnicity.

**Results:**

The groups that were further stratified into mild burn and severe burn had the following

**results:**

The mild burn risk ratio (RR) and 95% confidence interval (CI) for MI was (0.146/0.091-0.235), sepsis (0.662/0.48-0.914), and mortality was (0.433/0.305-0.615). The severe burn cohort had a RR and a 95% CI for MI of (0.236/0.121-0.462), sepsis (1.194/0.766-1.859), and mortality (0.547/0.416-0.719).

**Conclusions:**

The patients with earlier elevated cTI level within 72 hours had worse outcomes of MI and mortality in both severe and mild burns.

**Applicability of Research to Practice:**

The study indicates the cTI predictive potential which could be applied to monitor the high risk of complications in severe injured patients.

